# Neuroprotective effect of total flavonoids in stems and leaves of *Glycyrrhiza uralensis* Fisch. on oxidative stress in HT-22 cells and *Caenorhabditis elegans*

**DOI:** 10.18632/aging.204627

**Published:** 2023-04-04

**Authors:** Weijia Chen, Lei He, Hongyan Pei, Jianming Li, Yan Zhao, Ying Zong, Hong Kan, Zhongmei He, Rui Du

**Affiliations:** 1 College of Chinese Medicinal Materials, Jilin Agricultural University, Changchun 130118, China; 2 Key Laboratory of Animal Production, Product Quality and Security, Ministry of Education of China, Changchun 130118, China; 3 Jilin Provincial Engineering Research Center for Efficient Breeding and Product Development of Sika Deer of China, Changchun 130118, China

**Keywords:** *Glycyrrhiza uralensis* Fisch., *Caenorhabditis elegans*, neurodegenerative diseases, Alzheimer's disease, oxidative stress

## Abstract

The *Glycyrrhiza uralensis Fisch*. is a common traditional Chinese medicine. However, its aerial part is currently not widely studied and used. Therefore, we aimed to investigate the neuroprotective effects of total flavonoids in aerial stems and leaves of *Glycyrrhiza uralensis* Fisch. (GSF) by an *in vitro* LPS-induced HT-22 cell model and an *in vivo Caenorhabditis elegans* (*C. elegans*) model. In this study, cell apoptosis was evaluated by CCK-8 and Hoechst 33258 staining in LPS-induced HT-22 cells. Meanwhile, ROS level, mitochondrial membrane potential (MMP), and Ca^2+^ level were detected by the flow cytometer. *In vivo*, *C. elegans* was also investigated the effect of GSF on lifespan, spawning, and paralysis. Moreover, the survival of *C. elegans* to oxidative stimuli (juglone and H_2_O_2_), and the nuclear translocation of DAF-16 and SKN-1 were evaluated. The results showed that GSF could inhibit LPS-induced HT-22 cell apoptosis. Moreover, GSF decreased the levels of ROS, MMP, Ca^2+^, and malondialdehyde (MDA) and increased the activities of SOD and CAT in HT-22 cells. Furthermore, GSF did not affect the lifespan and laying of eggs of *C. elegans* N2. However, it delayed paralysis in *C. elegans* CL4176 in a dose-dependent manner. Meanwhile, GSF increased the survival rate of *C. elegans* CL2006 after juglone and H_2_O_2_ treatment, increased SOD and CAT, and decreased MDA levels. Importantly, GSF promoted the nuclear translocation of DAF-16 and SKN-1 in *C. elegans* TG356 and LC333, respectively. Taken together, GSF can play a protective role in neuronal cells by inhibiting oxidative stress.

## INTRODUCTION

Neurodegenerative diseases (ND) are one of the leading causes of mortality and morbidity worldwide, especially in older adults. Also, ND has received high and widespread attention with the increasing proportion of older individuals in society. The most commonly known neurodegenerative diseases are Alzheimer's disease (AD), Parkinson's disease (PD), Huntington's disease (HD), multiple sclerosis (MS), and amyotrophic lateral sclerosis (ALS) [1–3]. ND is known to occur when brain cells lose function and eventually die. Neurodegeneration results in progressive and irreversible loss of neurons and lead to brain atrophy [4]. Studies have shown that ND develops in different brain sites and exhibits different etiologies, and is associated with multiple links, including neuronal death, neuroinflammation, lysosome dysfunction, protein aggregation, DNA damage, etc. [5–8].

Notably, AD is a neurodegenerative disease, and the prevalence and incidence of AD increase with age. The latest data indicate that the population aged 65 or over is projected to increase from 9.3% in 2020 to around 16.0% in 2050 globally [9]. By 2050, the prevalence of dementia will double in Europe and triple globally [10]. It is characterized by memory loss and progressive neurocognitive dysfunction. AD not only affects personal and family life, but also brings a heavy burden to society. The neuropathological diagnostic criteria for AD are neurofibrillary tangles composed of extracellular aggregation of amyloid beta (Aβ) plaques and the associated protein tau in the cortical and limbic regions of the human brain [11, 12]. At present, the main pathogenesis of AD includes the Aβ toxicity hypothesis, cholinergic hypothesis, tau protein hypothesis, neuroinflammation hypothesis, mitochondrial dysfunction hypothesis, etc. Notably, studies have found that oxidative stress caused by abnormally increased production of reactive oxygen species (ROS) is closely related to AD in recent years [13, 14].

Oxidative stress, a key regulator in neurodegenerative diseases, has an important role in the process of neuronal apoptosis and death [15, 16]. Studies have shown that the brain is particularly vulnerable to oxidative stress because of its high oxygen consumption and weak antioxidant defenses [17]. When ROS levels in the brain increase, oxidative stress increases, which leads to mitochondrial dysfunction and is common in neurodegeneration [18, 19]. Furthermore, ROS-enhanced production may disrupt the redox balance of cells in the brain to an oxidative state, leading to its dysfunction and death [13]. Therefore, inhibiting the damaged antioxidant system may prevent or treat AD. Understanding the molecular mechanisms of oxidative stress may help improve memory impairment and treat AD disease.

Currently, treatments for AD cannot achieve satisfactory results, and only five drugs are approved by the FDA for treatment: donepezil, rivastigmine, galantamine, tacrine, and memantine. It suggests that finding new drugs to treat AD has become a top priority. *Glycyrrhiza uralensis* Fisch. (*G. uralensis.*) is known as a common Chinese medicinal herb and described in the 2020 edition of the Pharmacopoeia of the People‘s Republic of China. Its dried root and rhizome are often used for a variety of diseases. For example, it has anti-inflammatory, anti-tumor, anti-viral, anti-cancer, anti-diabetic, anti-oxidant, anti-ulcerative colitis, etc. [20–23]. Of course, the ethanol extract from *G. uralensis*. has been shown to inhibit nerve cell apoptosis and have a possible therapeutic role for preventing the progression of neurodegenerative disease such as Alzheimer's disease [24]. Its flavonoids are one of the active ingredients and have been extensively studied [25, 26]. Glycyrrhizic acid and isoglycyrrhizic acid can improve cognitive impairment in mice, and oxidative stress levels in *C. elegans* to play a role in the treatment of neurodegenerative diseases [27, 28]. However, the aerial part of *G. uralensis.* is not widely used, resulting in the waste of resources. Its aerial parts (stems and leaves) have high nutritional and medicinal value [26]. Studies have shown that the aerial parts of *G. uralensis*. are rich in flavonoids, including flavonoids, flavonols, flavonol glycosides, dihydroflavonoids, isoflavones, dihydroisoflavonoids, etc. [29]. Unfortunately, the pharmacological effects of stems and leaves of *G. uralensis.* (GS) are rarely reported.

In this study, the total flavonoids in GS (GSF) were used as the research object. We used HT-22 cells and *Caenorhabditis elegans* (*C. elegans*) as *in vitro* and *in vivo* models to elucidate the neuroprotective effects of GSF through inhibition of oxidative stress. It will provide the basic theory for the further development and research of GSF in the treatment of AD in the future.

## MATERIALS AND METHODS

### Materials

GS comes from Jilin Agricultural University, Changchun City, Jilin Province. The GSF was obtained under the following conditions [30]: ethanol concentration 75%, liquid-material ratio 20:1, ultrasonic time 60 min, temperature 50° C. After extraction with petroleum ether, the lower layer solution was eluted (70% ethanol) through an AB-8 macroporous resin column. Subsequent concentrated samples were eluted again (70% ethanol) through a polyamide resin column (pH=5 adjusted with glacial acetic acid). The dried sample obtained was GSF and contained about 70% of total flavonoids.

DMEM medium and fetal bovine serum (FBS) (Gibco, Australia); LPS, tetraethylbenzimidazolylcarbocyanine iodide (JC-1), Hoechst 33258 staining kit, Ca^2+^ indicator Fluo-3/AM, SOD, CAT, and MDA detection kit (Beijing Soleibo Technology Co., Ltd.); Cell counting kit-8 (CCK-8, Beijing Labgic Technology Co., Ltd.); Annexin V fitc PI (Shanghai Bioscience Technology Co., Ltd.); 2, 7-dichlorofluorescein diacetate (DCFH-DA) (Beyotime Biotechnology).

### *C. elegans* strains and HT22 cell

*C. elegans* strains N2 (wild type), CL4176 (dvls27 [myo-3p::A-Beta (1-42)::let-851 3’UTR+rol-6(su1006)]), CL2006 (dvls2 [Pcl12(unc-54/human Abeta peptide 1-42 minigene)+rol-6(su1006)]), LG333 (gels7 [skn-1b:GFP]), TJ356 (zIs356 [*daf-16p*::*daf-16a/b*:GFP + *rol-6*(*su1006*)] and *Escherichia coli* OP50 (*E. coli* OP50) were obtained from the Caenorhabditis Genetics Center (University of Minnesota, MN, USA). According to the standard protocol [31], all strains were cultured on nematode growth medium (NGM) containing live *E. coli* OP50 as food source at 20° C with the exception of CL4176 and CL2006 that was maintained at 16° C. For most experiments, *C. elegans* strains undergo development from the larval stage L1 to L4, which is considered the onset of adulthood, counted as adult day 0. Worms at the L4 stage were picked and transferred to start the experiment.

HT-22 cells are obtained from the Shanghai Gaining Biotechnology Co., Ltd.

### Concentration screening of GSF and LPS

HT-22 cells were grown in DMEM medium and 10% heat-inactivated FBS containing 100 U/mL penicillin and 100 μg/mL streptomycin. Cells were cultured in a carbon dioxide incubator (Thermo Fisher, USA) at 37° C and 5% CO_2_. HT-22 cells in the exponential phase of growth were used for all experiments. In this study, HT-22 cells were seeded onto a 96-well culture plate at a density of 5×10^4^ cells/well and grown at 37° C for 24 h. After that, the culture medium was discarded, and 100 μL of GSF solutions of different concentrations (5, 10, 25, 50 100, 250, and 500 μg/mL) and different concentrations of LPS (1, 2, 3, 4, and 5 μg/mL) was added to the cell culture, respectively. The control group contained only 10% FBS medium solution. After 24 hours, the culture medium was discarded. Moreover, the culture medium with 10 % CCK8 content was added to each well and cultured for 2 h according to the reagent manufacturer's instructions. The absorbance value was detected by a microplate reader (Epoch2; American Burton Instruments Co., Ltd., USA) at 450 nm. The cell viability (%) was calculated as follows: Cell viability (100%) = (A_drug_ - A_blank_) / (A_control_ - A_blank_) × 100%.

### Evaluation of LPS-induced cell viability with GSF

HT-22 cells were seeded in a 96-well plate at a concentration of 5 × 10^4^ cells/ml per well and cultured for 24 h. Subsequently, the culture medium was aspirated and discarded. The control group was added with 10% FBS medium, the model group was added with LPS concentration of 2 μg/mL, and the GSF group was added with GSF solution (25 and 50 μg/mL) and LPS solution (2 μg/mL). Then, the cell viability was calculated as above.

### Hoechst 33258 cell staining

HT-22 cells were seeded in 6-well plates at a concentration of 2.5 × 10^5^ cells/mL per well. Culture and drug treatments were performed as above. Cells are grouped as follows: control group, model group (LPS 2 μg/mL), and GSF group (25 and 50 μg/mL). After 24 h, cells were washed twice with pre-cooled PBS for 5 min each. The culture medium was discarded and 4% tissue cell fixative was added for 10 min. Then, washed twice with PBS and incubated with Hoechst 33258 staining solution for 20 min in the dark. After incubation, cells were washed twice with PBS. Fluorescence microscopy (Leica Microsystems CMS GmbH, Germany) was used to observe and photograph with an excitation wavelength of 350 nm and an emission wavelength of 460 nm.

### Measurement of SOD, CAT and MDA levels in HT-22 cells

HT-22 cells were collected from the above experiments to determine oxidative indicators, including SOD, CAT, and MDA. Assays were performed according to the respective manufacturer's protocol.

### Measurement of intracellular ROS

After HT-22 cells were treated as above, the cells were harvested. The density was 1×10^8^ cells/mL. Then, 10 μmol/L DCFH-DA was added to the cells and incubated at 37° C for 30 min. Afterward, the cells were washed twice with PBS and detected by Flow cytometry (BD FACSVerse, USA).

### Measurement of mitochondrial membrane potential (MMP)

HT-22 cells were collected after LPS-induced and drug treatment. The HT-22 cells were washed twice with PBS, and 1 mL of JC-1 staining solution and 1 mL culture medium were added and incubated at 37° C for 20 min. Subsequently, cells were washed twice and resuspended in 0.2 mL ice-cold PBS for analysis by Flow cytometry.

### Measurement of intracellular Ca^2+^

As described above, HT-22 cells were added with Ca^2+^ indicator Fluo-3/AM and incubated at 37° C for 20 min. Then, cells were washed three times with HEPES buffer saline to obtain a solution of 1×10^5^ cells/mL. After 10 min, samples were examined by Flow cytometry.

### Inhibitory effect of GSF on *E. coli* OP50

LB (Luria-Bertani) liquid cultures were formulated to contain different concentrations of GSF solutions (0, 50, 300, 600, 1200, 2400, 4800, and 6000 μg/mL). After that, inoculate 10% enriched single colony cultured liquid *E. coli* OP50. They will be co-incubated overnight in a 37° C water bath incubator. Then, 200 μL of the obtained samples were placed in 96-well plate (n = 6), and use a microplate reader to measure the absorbance at 500 nm. ΔA = A_sample_ - A_blank_.

### Evaluation of GSF on lifespan and egg laying in *C. elegans* N2

Thirty nematodes were picked on NGM containing GSF (0 μg/mL, 50 μg/mL, 300 μg/mL, 600 μg/mL) with *E. coli* OP50. The start of the experiment was defined as day 0 in the survival curve. Nematodes were scored as dead or alive every other day until all the animals died. In addition, the petri dishes need to be replaced every day after the nematodes enter the spawning period. In the spawning period, transfer each adult to a new plate and count all eggs and hatched larvae daily from the first day of spawning. The number of eggs laid was recorded daily. All the experiments were repeated three times independently.

### Paralysis assay of GSF to *C. elegans* CL4176

As mentioned above, thirty nematodes were picked on NGM containing the control group and GSF groups (50, 600, and 2400 μg/mL) with *E. coli* OP50. The nematodes were cultured to L3 stage in a constant temperature and humidity incubator at 16° C with a humidity of 50%-70%. Then, the nematodes were transferred to a constant temperature and humidity incubator at 25° C for 36 h. *C. elegans* CL4176 are paralyzed by elevated temperatures in the culture environment. The state of nematodes was observed and recorded for 8 h. They were rated as "paralyzed" when only small head movements were possible. All the experiments were repeated three times independently until all worms on each plate were paralyzed.

### Evaluation of GSF for oxidative stress survival in *C. elegans* CL2006

Synchronized *C. elegans* CL2006 was cultured in a constant temperature and humidity incubator at 16° C and 50%-70% humidity to L3 stage. The groups are as follows: control group and different GSF groups (50, 600, and 2400 μg/mL). Thirty nematodes were picked from each group and inoculated in NGM containing 500 mM juglone. Among them, the control group did not include juglone. Nematode survival was recorded every 2 hours.

Likewise, in the H_2_O_2_ oxidative stress experiment, *C. elegans* CL2006 was stimulated with 10 mM hydrogen peroxide, and the control group did not contain H_2_O_2_. The survival rate was recorded after 16 hours. All the experiments were repeated three times independently.

### Evaluation of SOD, CAT and MDA levels in *C. elegans* CL2006

The above four groups of synchronized *C. elegans* CL2006 were cultured in a constant temperature and humidity incubator at 16° C and humidity of 50%-70%. The nematodes were given GSF solutions (50, 600, and 2400 μg/mL) for 5 days after the L3 stage. Afterward, nematodes were collected in centrifuge tubes with M9 buffer. It was removed the supernatant, 1 mL of RIPA lysate was added, and was ultrasounded for 10 min. The supernatant was assayed according to the kit instructions.

### Evaluation of DAF-16 and SKN-1 nuclear translocation in *C. elegans* TJ356 and LG333

Age-synchronized transgenic nematodes, including theTJ356 strain that contained a DAF-16::GFP reporter gene and the LG333 strain that contained an SKN-1::GFP reporter gene. Synchronized TJ356 nematodes or LG333 nematodes were inoculated into petri dishes (=30 worms per worm) containing control group and GSF groups (50, 600, and 2400 μg/mL). After three days of incubation at 20° C, nematodes were collected and passed through M9 buffer to wash away residual *E. coli* OP50 from the nematodes. 10 mmol/L levamisole solution was used to anesthetize the nematodes. Subsequently, the GFP fluorescence of GFP-expressing populations was directly observed under a fluorescence microscope. To determine the rate of nuclear translocation, photographs were taken and analyzed using ImageJ software. All the experiments were repeated three times independently.

### Statistical analysis

All data are presented as mean ± SEM. Data were analyzed by one-way or ANOVA with Tukey’s test for comparison between multiple groups. A value of p < 0.05 was considered significant. Graphpad prism software was used for graphing.

## RESULTS

### Analysis of cell viability

**Figure 1:**
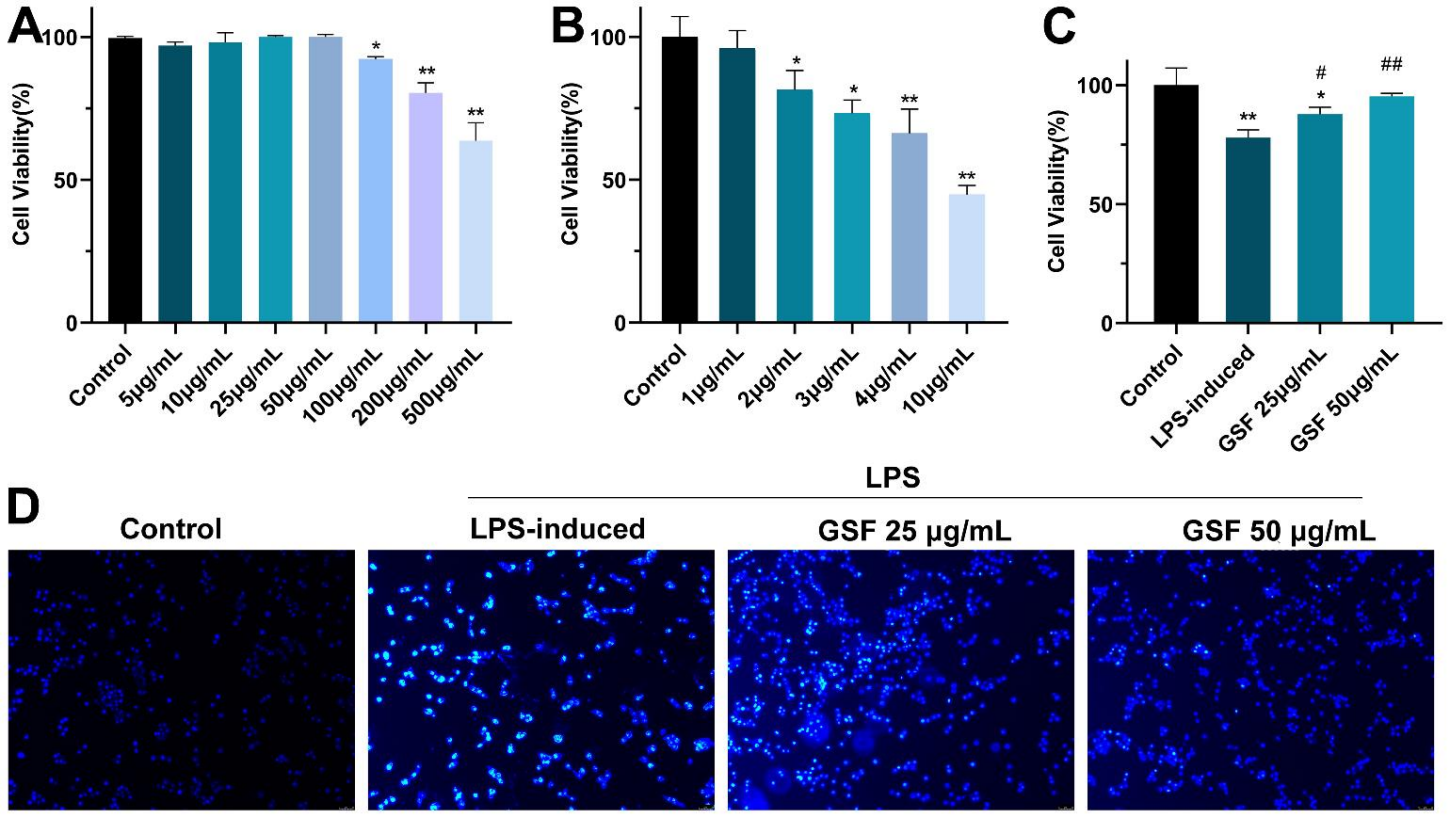
(**A**) The effect of GSF (5, 10, 25, 50 100, 250, and 500 μg/mL) on cell viability. (**B**) The effect of LPS (1, 2, 3, 4, and 5 μg/mL) on cell viability. (**C**) The effect of GSF on LPS-induced cell viability. (**D**) Hochest staining of HT-22 cell. *P<0.05, **P<0.01 was considered statistically significant compared with the control group; #P<0.05, ##P<0.01 was considered statistically significant compared with the LPS-induced group.

Here, the administration concentrations of GSF and LPS, and the protective effect of GSF on LPS-induced HT-22 cell injury were preliminarily evaluated by cell viability. Figure 1A shows the effect of GSF on cell viability. It showed that the 100 μg/ml, 250 μg/ml, and 500 μg/ml groups had significant inhibitory effects on cells (P<0.05 or P<0.01) compared with the control group. There was no significant difference between the 5-50 μg/mL group compared with the control group. Additionally, the effect of LPS on cell viability is shown in Figure 1B. The results showed that the toxic effect of LPS on HT-22 cells was in a dose-dependent manner. When cell viability is 81.48±5.45% (LPS concentration of 2 μg/mL) was used for subsequent experiments.

In subsequent experiments, the effect of GSF on LPS-induced HT-22 cell viability was evaluated. Figure 1C shows that GSF increased cell viability after LPS-induced, and cell viability was in a dose-dependent manner. It indicated that GSF could protect LPS-induced HT-22 cell damage.

### Hoechst 33258 cell staining analysis

Hoechst 33258 is a blue fluorescent dye that can penetrate the cell membrane and is often used to detect apoptosis. The healthy living cells exhibit uniform nuclear staining. Conversely, apoptotic cells exhibit nuclear pyknosis and fragmentation. As shown in Figure 1D, the cells in the control group were morphologically plump and cell membranes intact. In the LPS-induced group, there were apoptotic features, with a lot of blue fluorescent bright spots. Treatment with 25 μg/mL and 50 μg/mL GSF reduced the number of fluorescent bright spots. It indicated that GSF could effectively inhibit LPS-induced apoptosis in a dose-dependent manner.

### Effects of GSF on SOD, CAT and MDA of HT-22

**Figure 2:**
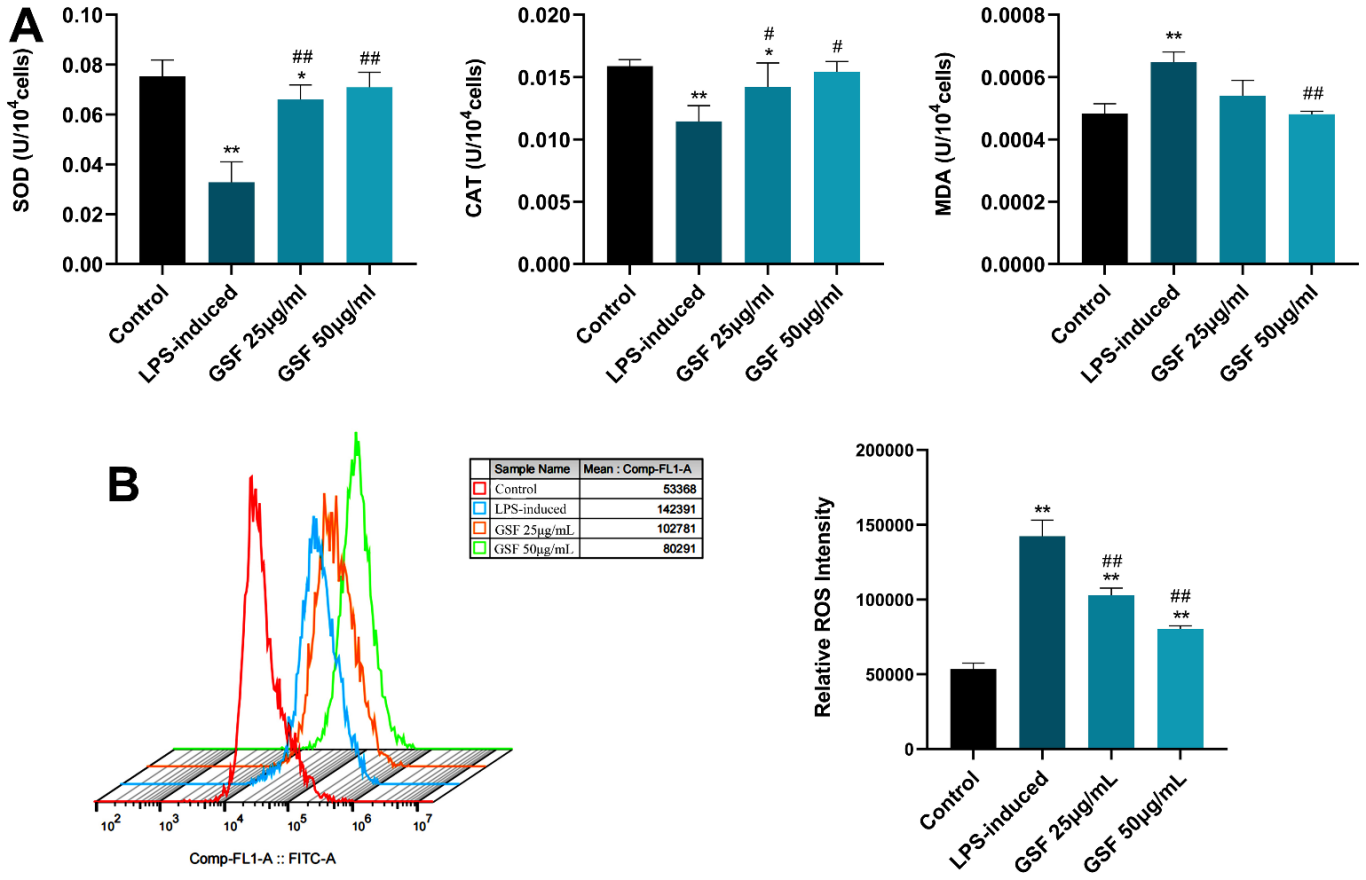
(**A**) Effects of GSF on SOD, CAT, and MDA levels in HT-22 cells. (**B**) ROS expression levels in HT-22 cells were detected by flow cytometry, and the relative ROS intensities were counted and displayed. *P<0.05, **P<0.01 was considered statistically significant compared with the control group; #P<0.05, ##P<0.01 was considered statistically significant compared with the LPS-induced group.

To test the protective effect of GSF on HT-22 cells subjected to oxidative stress, we assessed SOD production, MDA levels, and SOD activity, respectively. MDA is an oxidative biomarker, and a high MDA level indicate elevated oxidative stress. ROS-induced oxidative stress can be used as key indicators of CAT and SOD [32]. As shown in Figure 2A, GSF significantly increased the activities of SOD and CAT and significantly decreased the activities of MDA (P<0.05 or P<0.01). It suggests that GSF can effectively improve the relevant indicators of oxidative stress.

### Effects of GSF on ROS levels

ROS is key molecules of oxidative stress. Once ROS release increases, oxidative stress increases. Macromolecules in the mitochondrial structure are susceptible to oxidative damage, and the function of organelles is disrupted. Ultimately, this causes mitochondria to release cytochrome c and trigger apoptosis [33]. Next, we examined whether GSF-enhanced oxidative-stress resistance was due to its ROS-scavenging ability. The results are shown in Figure 2B. The level of ROS in the LPS-induced group increased significantly compared with the control group (P<0.01). After treatment with GSF, ROS levels were significantly inhibited (P<0.05). It suggests that GSF can reduce the level of ROS in HT-22 cells in a dose-dependent manner. Moreover, oxidative stress was suppressed by inhibiting ROS levels.

### Effects of GSF on LPS-induced MMP in HT-22 cells

**Figure 3:**
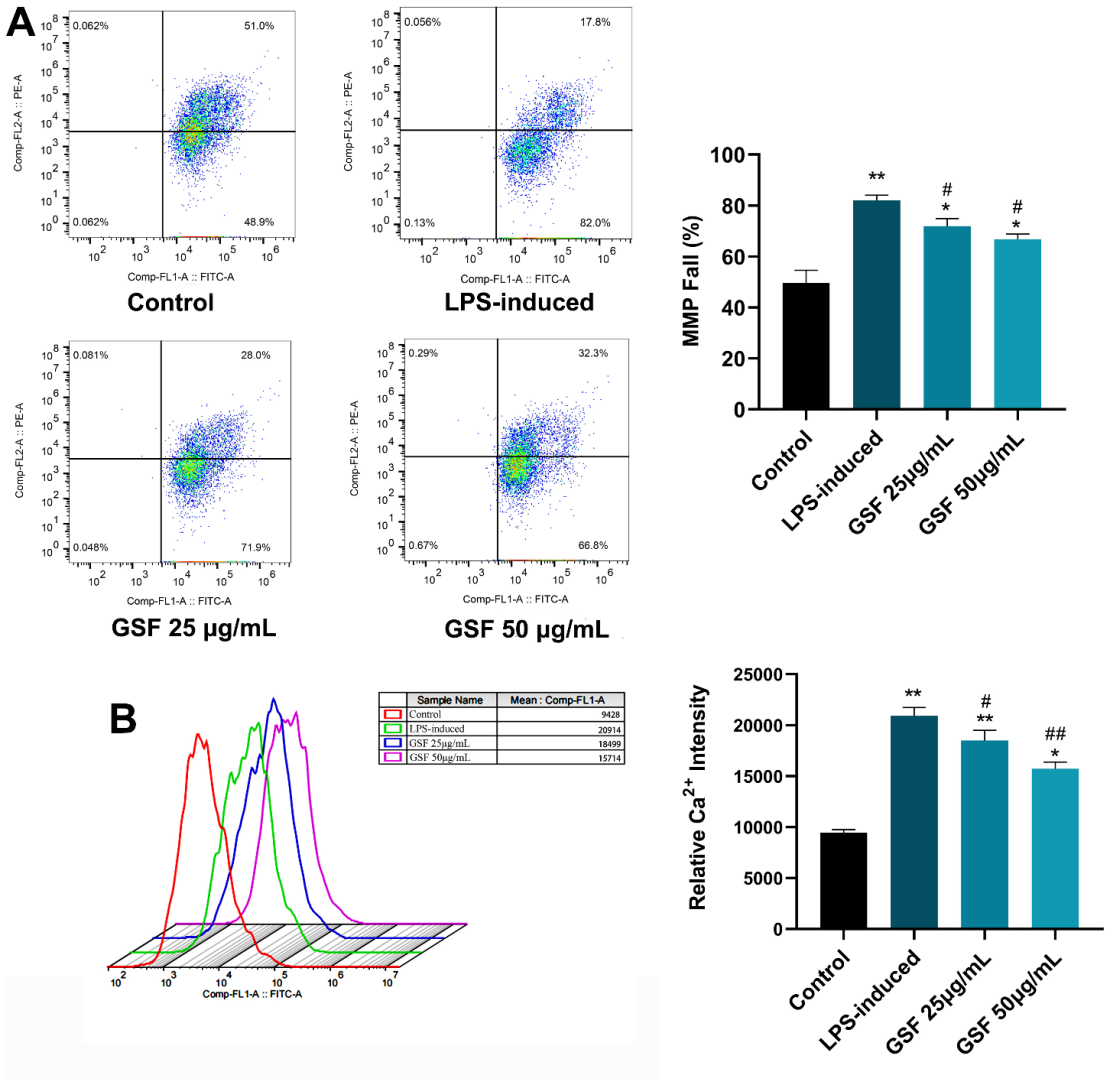
(**A**) MMP of HT-22 cells was determined using flow cytometry with JC-1 staining, and the MMP fall (%) were counted and displayed. (**B**) Ca^2+^ level of HT-22 cell after LPS-induced and GSF treatment was determined using flow cytometry, and the relative Ca^2+^ intensities were counted and displayed. *P<0.05, **P<0.01 was considered statistically significant compared with the control group; #P<0.05, ##P<0.01 was considered statistically significant compared with the LPS-induced group.

When oxidative stress occurs, ROS levels increase, leading to mitochondrial dysfunction. Mitochondrial dysfunction, in turn, leads to reduced ATP production, dysregulated Ca^2+^ balance, and ROS production [34]. Therefore, MMP was evaluated to investigate the regulatory effect of GSF. As shown in Figure 3A, the MMP decreased rate in the LPS-induced group was significantly increased compared with that in the control group (P<0.01). In contrast, the mitochondrial membrane potential of the GSF group was significantly decreased compared to the LPS-induced group. Additionally, the 50 μg/mL group was superior to the 25 μg/mL group. It indicates that GSF can ameliorate LPS-induced MMP increase.

### Effects of GSF on intracellular Ca^2+^ levels

Mitochondrial dysfunction, excessive ROS production affects the pathogenesis of AD, resulting in mitochondrial Ca^2+^ dysregulation, loss of ATP [31]. Here, Ca^2+^ levels were evaluated by flow cytometry. As shown in Figure 3B, Ca^2+^ levels were significantly increased (P<0.01) in the LPS-induced group. Interestingly, Ca^2+^ levels were decreased after GSF treatment. It suggests that GSF can protect HT-22 cells by improving mitochondrial Ca^2+^ dysregulation.

### Inhibitory effect of GSF on *E. coli* OP50

**Figure 4:**
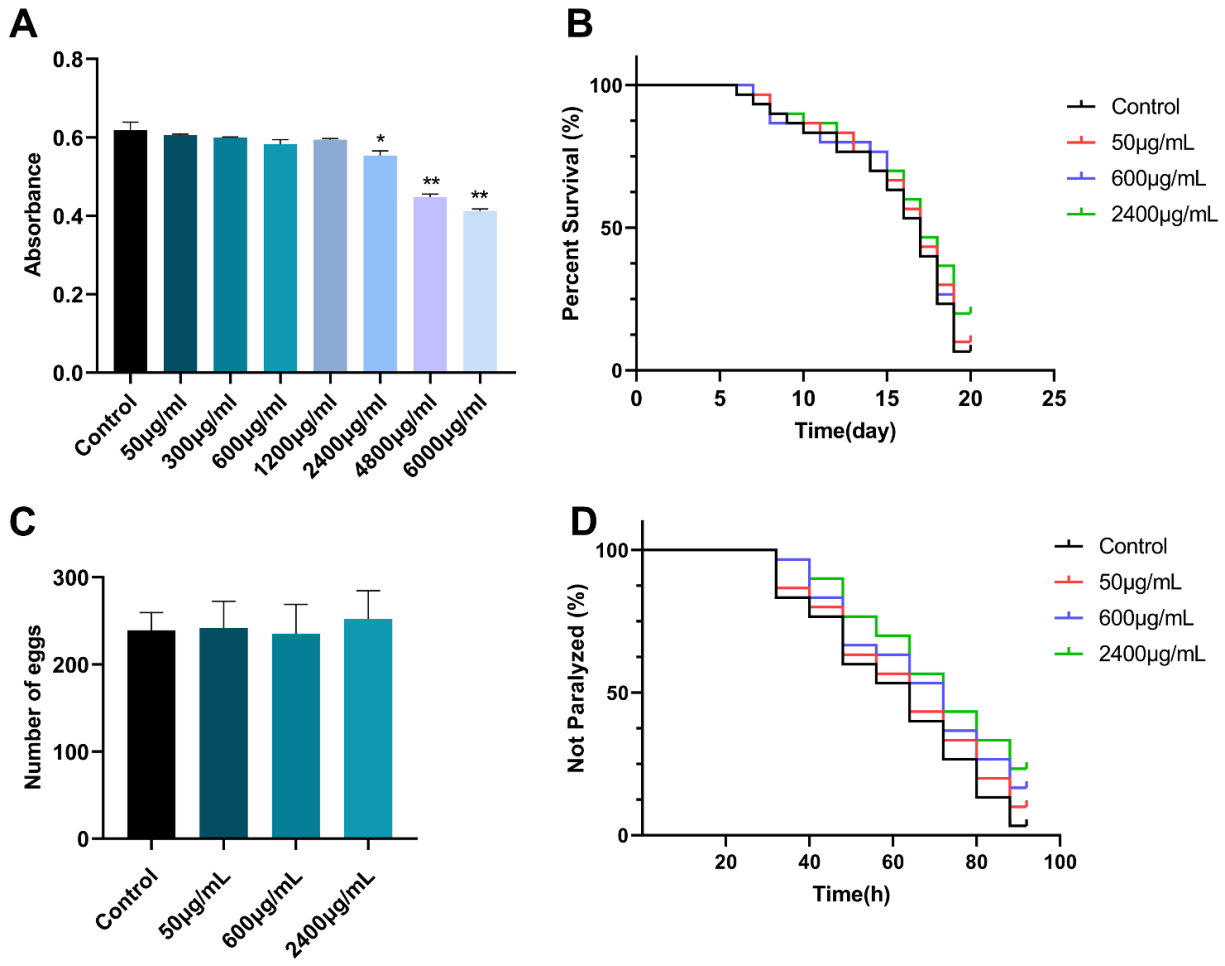
(**A**) The effect of GSF on E. coli OP50 is represented by absorbance values. (**B**) The effect of GSF on the lifetime of *C. elegans* N2. (**C**) The effect of GSF on the number of eggs laid in *C. elegans* N2. (**D**) The effect of GSF on the paralysis of *C. elegans* CL4176. *P<0.05, **P<0.01 was considered statistically significant compared with the control group.

To investigate the effect of GSF on *E. coli* OP50 of nematode food and the effect of food intake on nematodes. Different concentrations of GSF were added to *E. coli* OP50 and absorbance values were measured. As shown in Figure 4A, compared with the control group, there were significant differences between 6000 μg/mL and 4800 μg/mL. It shows that GSF concentration ≤2400 μg/mL has no effect on the growth of *E. coli* OP50. Therefore, the effect of food intake on *C. elegans* was excluded when the GSF concentration was ≤2400 μg/mL.

### Effects of GSF on lifespan and oviposition in *C. elegans* N2

To investigate the effect of GSF on the lifespan and oviposition of *C. elegans*, synchronized N2 worms were subjected to GSF at various concentrations (50, 600, and 2400 μg/mL). As shown in Figure 4B, 4C, GSF had no significant effect on C. elegans N2 lifespan and egg laying number (P>0.05). It revealed that concentrations of GSF ≤ 2400 μg/mL had no significant toxic effects on lifespan and oviposition.

### Effects of GSF on the paralytic phenotype of *C. elegans* CL4176

The main pathological change in the paralysis of *C. elegans* is the massive deposition of Aβ, which causes senile neurodegeneration and is associated with AD [35]. To determine whether GSF protects against Aβ-induced toxicity, we evaluated the effect of GSF on delaying Aβ-induced paralysis in transgenic *C. elegans* CL4176. The results showed that we performed paralysis tests on CL4176 worms using GSF solutions (50, 600, and 2400 μg/mL). It delayed paralysis in *C. elegans* CL4176 in a dose-dependent manner (Figure 4D). The result suggests that GSF has a potential therapeutic effect on AD.

### Effects of GSF on oxidative stress in *C. elegans* CL2006

**Figure 5:**
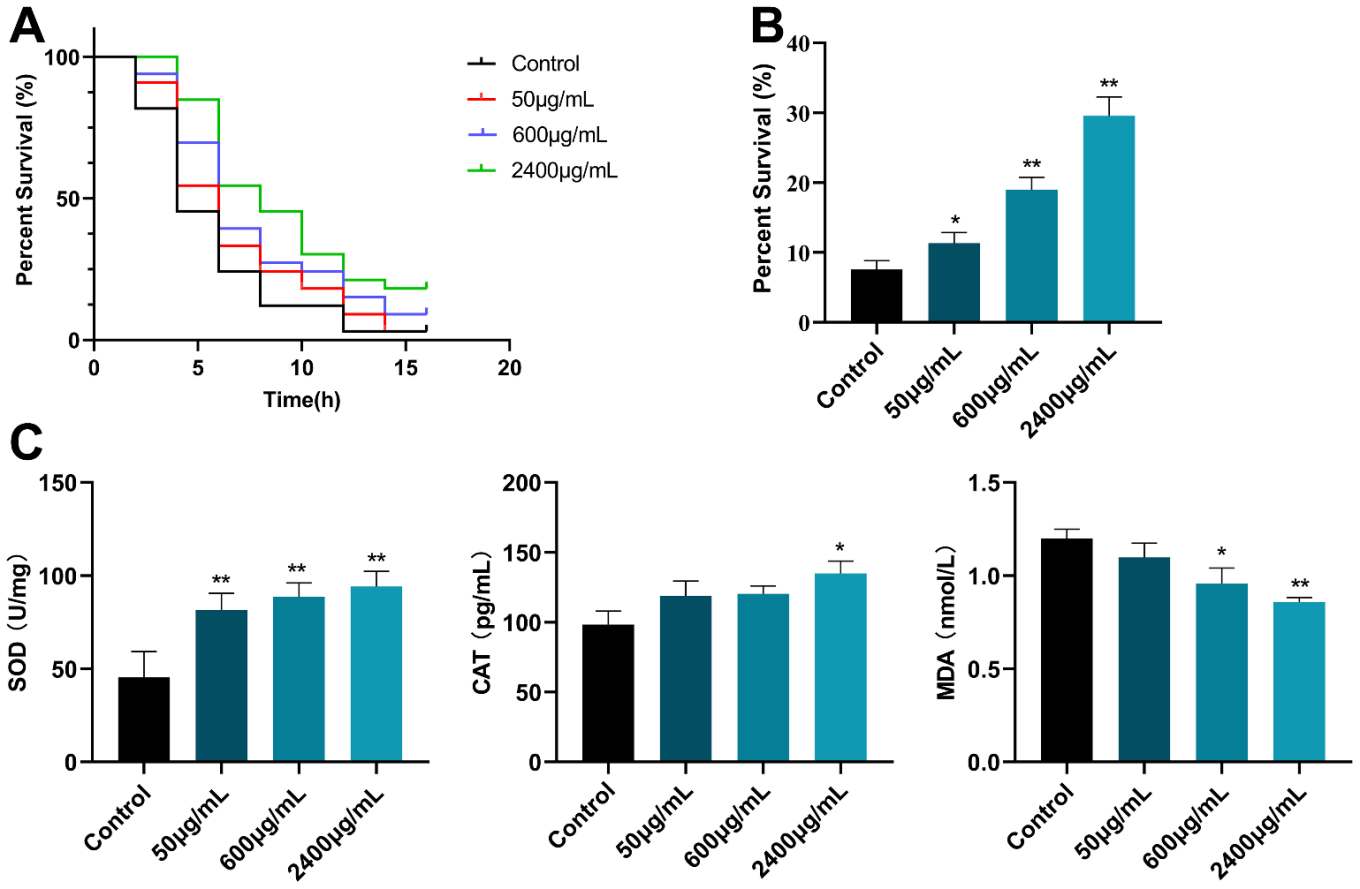
(**A**) Survival curve of *C. elegans* CL2006 after juglone stimulation. (**B**) Survival of *C. elegans* CL2006 after H_2_O_2_ stimulation. (**C**) The content of SOD, CAT, and MDA in *C. elegans* CL2006. *P<0.05, **P<0.01 was considered statistically significant compared with the control group.

Here, we examined the nematode responses to oxidative stimuli and the protective effects of GSF. Juglone, as one of the commonly used strong oxidants, can release a large amount of ROS and shorten the lifespan of the body. As shown in Figure 5A, compared with control, the GSF (50, 600, and 2400 μg/mL) group significantly prolonged the survival rate of nematodes after juglone oxidative damage (P<0.05 or P<0.01), and was positively correlated with the dose. The results showed that treatment with GSF significantly increased the survival of worms exposed to juglone-induced oxidative stress.

Likewise, the oxidative stress assay was further evaluated by using H_2_O_2_. H_2_O_2_, a commonly used bleach and detergent, can produce a lot of reactive oxygen species, consume a lot of energy and cause the body to age. As shown in Figure 5B, GSF (50, 600, and 2400 μg/mL) significantly increased the survival time of nematodes (P<0.05 or P<0.01) compared to the control (0 μg/mL). In agreement with the observation by juglone, GSF significantly increases survival after H_2_O_2_ exposure.

Additionally, as shown in Figure 5C, GSF significantly increased the activities of SOD and CAT in nematodes and significantly decreased the activities of MDA in nematodes (P<0.05 or P<0.01). The above results indicate that GSF has an excellent antioxidant effect.

### Effects of GSF on nuclear translocation of DAF-16 and SKN-1

**Figure 6:**
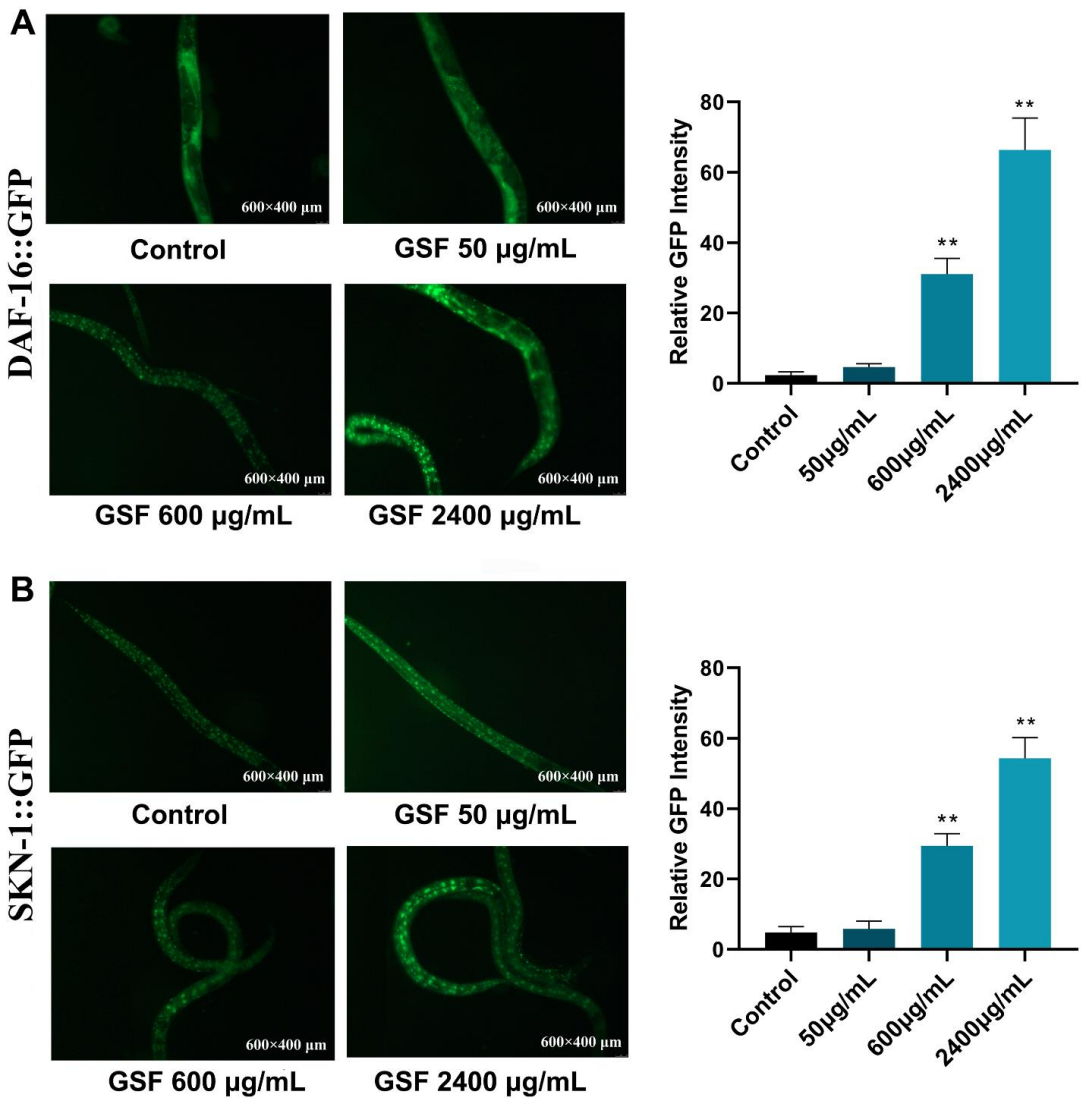
(**A**) Fluorescence images of DAF-16 nuclear localization and the quantification of DAF-16 nuclear localization. (**B**) Fluorescence images of SKN-1 nuclear localization and the quantification of SKN-1 nuclear localization. *P<0.05, **P<0.01 was considered statistically significant compared with the control group.

To identify the GSF mechanism of action, we examined its effect on DAF-16 and SNK-1. DAF-16 is the only forkhead box transcription factors class O (FoxO) homolog, and SKN-1is the ortholog of mammalian Nrf proteins in *C. elegans*. They are well-known longevity factors and play a key role [36, 37]. As shown in Figure 6A, when the GSF concentration was ≤50 μg/mL, TG356 nematodes exhibited uniform green fluorescence all over the body. However, at GSF concentrations of 600 and 2400 μg/mL, the fluorescence was distributed in punctate form. It indicates that DAF-16 is activated and enters the nucleus from the cytoplasm, and nuclear translocation occurs. And DAF-16::GFP nuclear translocation was positively correlated with dose. Figure 6B evaluates SKN-1::GFP nuclear translocation by GSF in *C. elegans* LC333, and the results are consistent with the above. The 600 μg/mL and 2400 μg/mL groups showed evident punctate fluorescence in the nematode intestine, and showed a dose-dependent manner. It indicated that SKN-1 was activated and nuclear translocation occurred. Consequently, our results illustrated that the longevity-promoting effect of GSF is dependent on DAF-16 and SKN-1. Moreover, the results suggest that GSF enhances oxidative stress resistance in *C. elegans* via DAF-16 and SKN-1.

## DISCUSSION

Oxidative stress is an imbalance between oxidants and antioxidants in the body. It leads to an increase in ROS levels and the activation of related oxygen free radicals. And these oxidative free radicals can promote the degeneration of neuronal cells [38]. Oxidative imbalance in neuronal damage may play a central role in AD [16]. Therefore, we assessed oxidative stress in an *in vitro* LPS-induced HT-22 cell model, and in an *in vivo C. elegans* model, and treated with GSF in this study.

*In vitro* experiments, LPS was used to induce inflammatory manifestations in HT-22 cells, as recurrent inflammation is closely associated with AD. Certainly, research reports suggest that LPS can be used to model AD. LPS in the human brain and abundant LPS in the AD brain are associated with amyloid plaques, perivascular amyloid, and neurons [39]. Moreover, mitochondrial dysfunction and oxidative stress is a significant feature in the pathomechanism of AD. Mitochondrial dysfunction leads to mitochondrial membrane depolarization, mitochondrial swelling, and MMP disturbances, together with apoptosis, disruption of Ca^2+^ homeostasis, inflammation, oxidative stress, and insufficient glucose metabolism [40]. Additionally, the accumulated potentially toxic level of ROS and Ca^2+^ is released from the mitochondria in time. Conversely, the released ROS in turn accelerate mitochondrial dysfunction and cellular damage [41]. Likewise, ND is associated with the production and accumulation of ROS, a critical factor in oxidative stress. An imbalance between ROS production and its oxidation is triggered, which affects mitochondrial respiratory chain function, thereby altering membrane permeability and calcium homeostasis [42]. In this experiment, we found that GSF could inhibit LPS-induced apoptosis, and decrease ROS, MMP, and Ca^2+^ levels. It suggests that GSF can protect nerve cells by inhibiting the oxidative stress pathway.

After, we used *C. elegans* as an *in vivo* model to examine the protective potential and anti-oxidative stress effects of GSF. *C. elegans* is used as a powerful tool for studying neuroprotective compounds. Despite their short lifespan, *C. elegans* shares many conserved molecular pathways and cellular mechanisms with mammals [43]. Importantly, it has 3357 worm genes with human orthologs. Additionally, the adult *C. elegans* has approximately 300 neurons throughout the body, reducing the complexity and increasing the accuracy of neuronal analysis. It is an excellent model for screening and evaluating ND [44]. In the present study, we found that treatment with GSF significantly increased the survival of *C. elegans* under oxidative stress (juglone or H_2_O_2_)-induced oxidative stress, indicating that GSF has antioxidant activity. Subsequently, to determine the mechanism of action of GSF, we examined its effect on the translocation of DAF-16 and SKN-1 from the cytoplasm to the nucleus. The results showed that GSF could enhance the translocation of DAF-16 and SKN-1 from the cytoplasm to the nucleus with increasing concentration.

In conclusion, our study demonstrates for the first time that GSF protects nerve cells by inhibiting oxidative stress. These findings suggest that GSF may act as an anti-oxidative stress agent. In addition, GSF may protect against oxidative stress-related diseases such as AD. Therefore, in the future research, the pharmacological mechanism of GSF should be further studied, including the beneficial effects of GSF in experiments of various neurodegenerative diseases, with a view to translating into the clinic.
